# Software-based risk stratification of pulmonary adenocarcinomas manifesting as pure ground glass nodules on computed tomography

**DOI:** 10.1007/s00330-017-4937-2

**Published:** 2017-07-14

**Authors:** Ursula Nemec, Benedikt H. Heidinger, Kevin R. Anderson, Michael S. Westmore, Paul A. VanderLaan, Alexander A. Bankier

**Affiliations:** 1 Department of Biomedical Imaging and Image-guided Therapy, Vienna General Hospital, Medical University of Vienna, Waehringer Guertel 18-20, Vienna, Austria; 2 Radiology, Beth Israel Deaconess Medical Center, Harvard Medical School, Boston, MA USA; 3 Pathology, Beth Israel Deaconess Medical Center, Harvard Medical School, Boston, MA USA; 4 Imbio, Delafield, WI USA

**Keywords:** Lung adenocarcinoma, Pure ground glass nodule, Risk stratification, Computed tomography, Software based

## Abstract

**Objectives:**

To assess the performance of the “Computer-Aided Nodule Assessment and Risk Yield” (CANARY) software in the differentiation and risk assessment of histological subtypes of lung adenocarcinomas manifesting as pure ground glass nodules on computed tomography (CT).

**Methods:**

64 surgically resected and histologically proven adenocarcinomas manifesting as pure ground-glass nodules on CT were assessed using CANARY software, which classifies voxel-densities into three risk components (low, intermediate, and high risk). Differences in risk components between histological adenocarcinoma subtypes were analysed. To determine the optimal threshold reflecting the presence of an invasive focus, sensitivity, specificity, negative predictive value, and positive predictive value were calculated.

**Results:**

28/64 (44%) were adenocarcinomas in situ (AIS); 26/64 (41%) were minimally invasive adenocarcinomas (MIA); and 10/64 (16%) were invasive ACs (IAC). The software showed significant differences in risk components between histological subtypes (P<0.001–0.003). A relative volume of 45% or less of low-risk components was associated with histological invasiveness (specificity 100%, positive predictive value 100%).

**Conclusions:**

CANARY-based risk assessment of ACs manifesting as pure ground glass nodules on CT allows the differentiation of their histological subtypes. A threshold of 45% of low-risk components reflects invasiveness in these groups.

***Key points*:**

• *CANARY-based risk assessment allows the differentiation of their histological subtypes.*

• *45% or less of low-risk component reflects histological invasiveness.*

• *CANARY has potential role in suspected adenocarcinomas manifesting as pure ground-glass nodules.*

**Electronic supplementary material:**

The online version of this article (doi:10.1007/s00330-017-4937-2) contains supplementary material, which is available to authorized users.

## Introduction

The “Computer Aided Nodule Assessment and Risk Yield” (CANARY) software package has recently been introduced for risk stratification of pulmonary nodules of the lung adenocarcinoma spectrum [1]. CANARY is based on assessing voxel densities of pulmonary nodules and the subsequent assignment of risk, as inferred from histology, according to voxel proportion and clustering. While this software has been successful in assessing both histology and outcome in a representative but morphologically diverse number of lung adenocarcinomas (AC) [1–3], it has not yet been tested in lung adenocarcinomas with morphologically more uniform appearance on computed tomography (CT).

A distinct subgroup of lung adenocarcinomas with a more uniform CT appearance, are those manifesting as pure ground glass nodules. They are considered to reflect a specific subgroup of adenocarcinomas with tailored management recommendations according to the ‘Guidelines for Management of Incidental Pulmonary Nodules Detected on CT Images: From the Fleischner Society 2017 [4]. These adenocarcinomas manifesting as pure ground glass nodules are important because they are common and can represent different histologic subtypes, ranging from noninvasive adenocarcinoma in situ (AIS) to invasive subtypes such as minimally invasive ACs (MIAs) and invasive ACs (IACs) [5]. A tool to differentiate among these histological subtypes in lung ACs could help to reduce diagnostic uncertainty in the management of such nodules. Therefore, the specific purpose of our study was to investigate the performance of CANARY software in the differentiation and risk assessment of histological subtypes of lung adenocarcinomas manifesting as pure ground glass nodules on CT.

## Materials and methods

The protocol for this retrospective study (#15-020) was approved by our institutional review board with the need for written informed consent waived.

### Study population

This retrospective study was based on a radiology-pathology data repository, which has served for other studies in the past, with a partial overlap of lesions and patients [6–8]. We reviewed the medical records of patients undergoing surgical resection for primary pulmonary adenocarcinoma (AC) at our institution between January 2005 and July 2016. Inclusion criteria were: (1) histologically verified primary pulmonary AC; (2) available pre-operative computed tomography (CT) examination; and (3) AC manifesting as pure ground glass nodule (GGN) on CT. Exclusion criteria were (1) histologic diagnosis other than AC; (2) pre-operative CT examination not available; (3) AC manifesting as a solid or part-solid nodule on CT; and (4) pathologically described nodule not identifiable on CT.

The medical record review resulted in 698 resected ACs, of which 89 (12.4%) were excluded because pre-operative CT examinations were not available. After further radiologic consensus review by two thoracic radiologists (AAB and BHH, with 20 and 2 years of experience, respectively), 546/611 (89.4%) adenocarcinomas were excluded because either the nodules were not identifiable on CT or they manifested as solid or part-solid lesions. Thereafter, the surgical resected specimens of the 65 remaining ACs were re-reviewed by a subspecialty-trained thoracic pathologist (PAV) and a senior pathology resident (KRA). This histopathological consensus review excluded 1/65 (1.5%) nodule because it was an atypical adenomatous hyperplasia (AAH). This left 64 adenocarcinomas manifesting as pure GGNs for CANARY analysis.

### CT acquisitions

CT examinations were performed using various CT scanner units and acquisition protocols, which were all considered state-of-the-art at the time of acquisition. The most frequently used CT units were Aquilion One (Toshiba, 320-detector row), Discovery CT750 HD (GE Medical Systems, 64-detector row), and Lightspeed VCT (GE Medical Systems, 64-detector row).

All CT examinations were performed in the supine body position, at full inspiration, covering the entire lung. Examinations before April 2007 were performed with fixed mAs (range: 130-340 mAs) and 120 kVp. After April 2007, automated exposure control and other dose reduction algorithms were used. Transverse images were reconstructed with 0.625 to 1.5 mm section thickness using standard reconstruction kernels in lung window settings (mean, −500 HU; width, 1500 HU). CT examinations for staging purposes (*n*=21, 36%) were performed using intravenous contrast material. In the remaining 38 (64%) examinations, no contrast material was administered.

### CT

The CT examinations were anonimised and presented in a random order on a picture archiving and communication system (GE Healthcare, Centricity) to two thoracic radiologists independently. Both radiologists were unaware of clinical and histological information. The long-axis and short-axis diameters of all 64 ACs were measured. Measurements were performed in lung window setting on the transverse CT section that displayed the largest nodule dimensions. Measurements were recorded in millimetres. Average CT diameters were calculated based on the long- and short-axis diameter.

### Computer Aided Nodule Assessment and Risk Yield (CANARY)

**Fig. 1:**
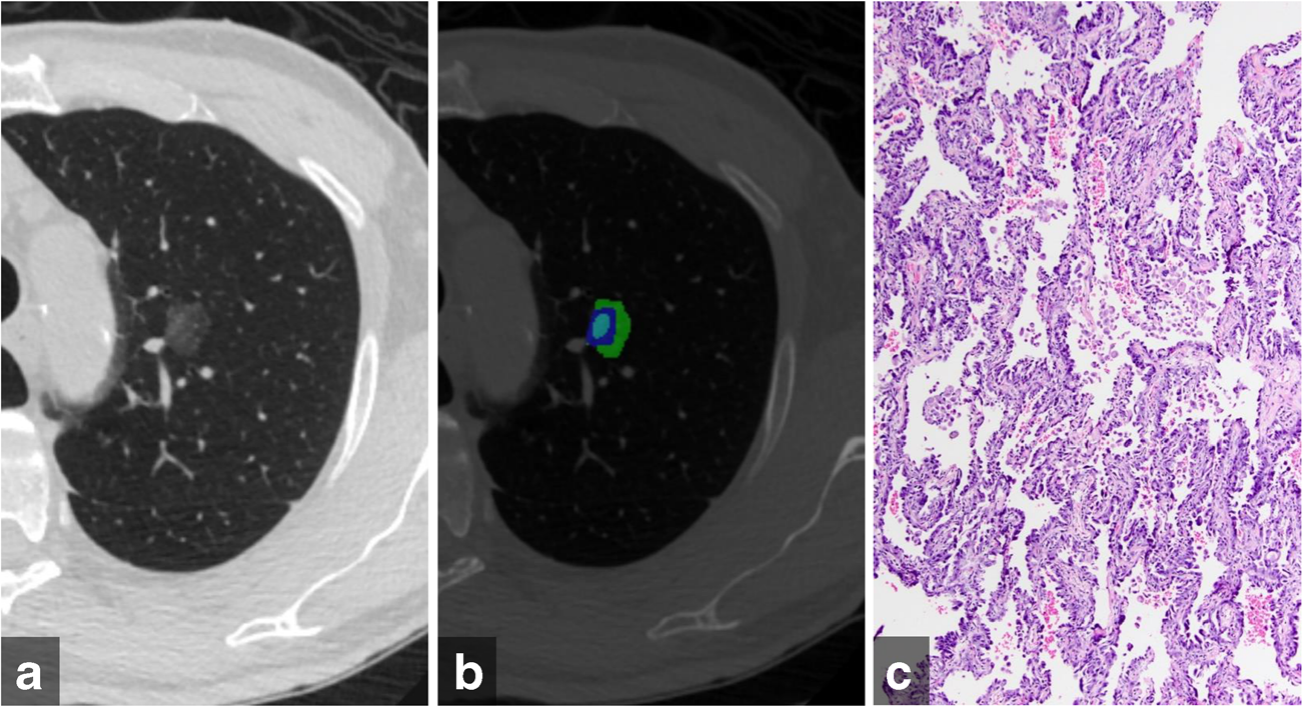
(a) Transverse computed tomography image of an adenocarcinoma in situ manifesting as pure ground glass nodule in the left upper lobe (b) Color-coded CANARY output overlay shows components of low risk group (blue-green–cyan) (low risk 99.8%, intermediate risk 0.2%, high risk 0%) (c) histologic image showing purely lepidic tumour growth pattern without areas of invasion (H&E stain, 200x original magnification)

All included nodules were also analysed by CANARY, a software for automated risk assessment of ACs based on the reduction of voxel density histograms to nine natural clusters. In a pilot study, these clusters were identified by selecting 774 regions of interest (two dimensional area ROIs, 9 x 9 voxels) in 37 ACs and comparing all ROIs to one another using Affinity Propagation and pairwise similarity metrics [1]. Exemplars were generated from these nine natural clusters and color-coded as violet (V), indigo (I), blue (B), green (G), yellow (Y), orange (O), red (R), cyan (C) and pink (P). These exemplars were then used for allocation of nodule risk components. To allocate nodule risk components, the 9 x 9 region around each voxel within the segmented nodule is compared to these nine exemplars to determine which cluster the voxel is most similar to. This results in a specific combination of colour codes for each nodule. A representative nodule is shown in Fig. 1.

For lung nodule characterisation, all 64 GGNs were located by one observer (UN, 4 years of experience in general radiology) using OsiriX (v8.0.1, Pixmeo SARL, Bernex, Switzerland). Initial data processing steps involved the segmentation of the nodule with the brush tool and pre-determined pixel values for inside and outside the ROIs. Predetermined pixel-values were arbitrarily defined numbers required by the CANARY algorithm to determine what is inside or outside of the ROI. ROIs were then saved as DICOMs and uploaded for CANARY analysis. Time needed for CANARY analysis averaged 10 minutes per lesion.

For each nodule, the CANARY analysis yielded the absolute and relative volume of each colour. These nine colours were then combined into three components as determined by Maldonado et al. [1]: (1) low risk – blue-green-cyan; (2) intermediate risk – pink-yellow; and (3) high risk – violet-indigo-red-orange.

The absolute and relative volumes of the three components of each nodule were then used for analysis.

### Histologic Review

The original histologic glass slides from all 64 resected nodules were retrieved and re-reviewed by a subspecialty-trained thoracic pathologist (PAV, 5 years of experience) and a senior pathology chief resident (KRA, PGY4). The histologic growth patterns (including the non-invasive/inferred *in-situ* lepidic pattern as well as any invasive patterns including acinar, papillary, micropapillary, and solid growth) were recorded in semiquantitative 5% increments according to current practice recommendations [9]. The size and number of any invasive foci (by definition, any histologic pattern other than lepidic) were assessed. Based on this information, these adenocarcinomas were classified using current WHO terminology and diagnostic criteria as AIS, MIA, or IAC [9]. AIS is defined as a solitary lung adenocarcinoma ≤3.0 cm demonstrating a pure lepidic growth pattern without evidence of stromal, vascular, or pleural invasion, tumour necrosis, or spread of tumour through the airspaces. MIA is defined as a solitary lung adenocarcinoma ≤3.0 cm with a predominantly lepidic growth pattern but that contains an invasive focus (i.e., growth pattern other than lepidic) that is ≤0.5 cm in greatest dimension, and also lacks lymphatic, vascular, or pleural invasion, tumour necrosis, or spread of tumour through the airspaces. In contrast, IAC are lung adenocarcinomas that either are greater than 3.0 cm, have an invasive growth pattern greater than 0.5 cm, or that demonstrate any of the more aggressive features lacking in AIS or MIA (lymphatic, vascular, or pleural invasion, tumour necrosis, or spread of tumour through the airspaces). Any discrepancies in tumour component measurement or classification between PAV and KRA were resolved via multihead microscope consensus review.

### Statistical analysis

All statistical analyses and graphs were performed using commercially available software (STATA 12.0, StatCorp, College Station, TX, USA). The normality of distributions was assessed by the Shapiro-Wilk test. Normally distributed data were expressed as mean ± SD. Non-normally distributed data were expressed as median together with their [interquartile range]. Statistical Power Analysis was performed using STATA module for simulation-based power analysis. A *p* value less than 0.05 was considered statistically significant.

First, we calculated the distribution of AIS, MIA, and IACs of the 64 GGNs included in this study.

Second, we calculated the medians of the low risk, intermediate risk, and high risk CANARY components for AIS, MIA, and IACs, respectively. Potential differences between the CANARY components in the histological subtypes, namely AIS, MIAs and IACs, were assessed for statistical significance using a Kruskal-Wallis test, with individual differences additionally assessed with Conover-Iman tests.

Third, we tested the strength of the relation of the size of invasive foci with both volume and percentage of the three CANARY components by calculating Spearman-rank correlation coefficients. All *P*-values obtained from Spearman correlation were Bonferroni corrected.

Fourth, to assess the presence of invasive foci, we grouped the two histological components with an invasive component, namely MIAs and IACs, and plotted individual percentages of the low risk and intermediate risk group, respectively.

Based on those individual percentages, we defined threshold values for low risk group in intervals of five percentages starting at 40%. To determine the optimal threshold reflecting the presence of an invasive focus, we calculated sensitivity, specificity, negative predictive value (NPV), and positive predictive value (PPV) for each threshold value.

To assess the influence of contrast material administration on the CANARY analysis, we performed a linear regression analysis with CANARY components as dependent variables and contrast administration and section thickness as independent variables.

## Results

The 64 ACs manifesting as pure GGNs on CT were detected in 59 patients, 5/59 (8%) of whom had more than one resected GGN, but no patient had more than two resected nodules.

There were 40/59 (67.8%) women (mean age 67±9 years; range, 45-84 years), and 19/59 (32.2%) men (mean age 69±9 years; range, 49-86 years). There was no significant difference in age between women and men (*P*=0.439). The median duration between the CT examination and surgical resection was 1 month [IQR 0-2].

GGNs were located as follows: right upper lobe (22; 34%); left upper lobe (18; 28%); right lower lobe (13; 20%), left lower lobe (9; 14%) and the right middle lobe (2; 3%).

Twenty-five (39%) of the nodules were removed by lobectomy, 28 (44%) by wedge resection, and 11 (17%) by segmentectomy.

Using an alpha level of 0.05, retrospective power analysis showed a statistical power of 0.95 for low risk components, 0.94 for intermediate risk component and 0.55 for high risk component.

Of the 64 GGNs included in our study, 28 (44%) were AIS, 26 (41%) were MIAs, and 10 (16%) were IACs. The average CT diameter of the nodules was 14.4± 5.3 mm.

**Fig. 2:**
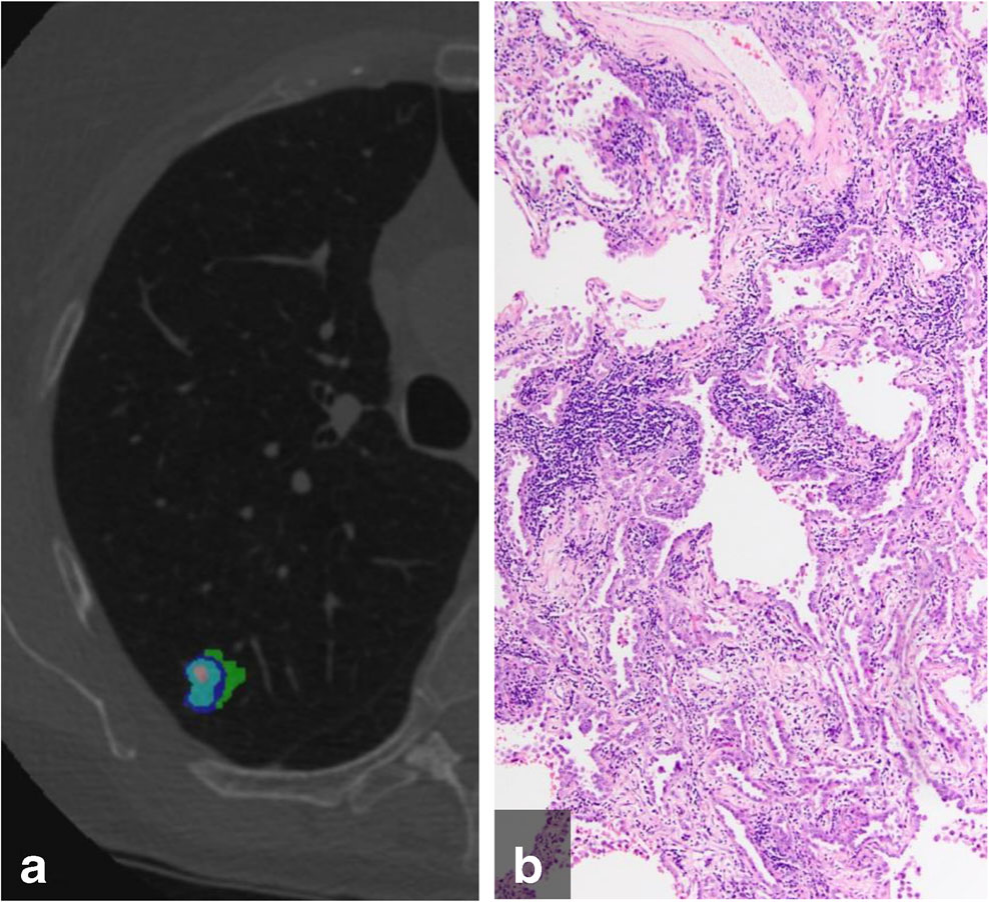
(a) Color-coded CANARY output overlay of adenocarcinoma in situ (blue-green-cyan-pink) (low risk 94.9%, intermediate risk 5.1%, high risk 0%);. (b) histologic image showing patchy interstitial chronic inflammation, but a purely lepidic tumour grown pattern without areas of invasion (H&E stain, 100x original magnification)

**Fig. 3:**
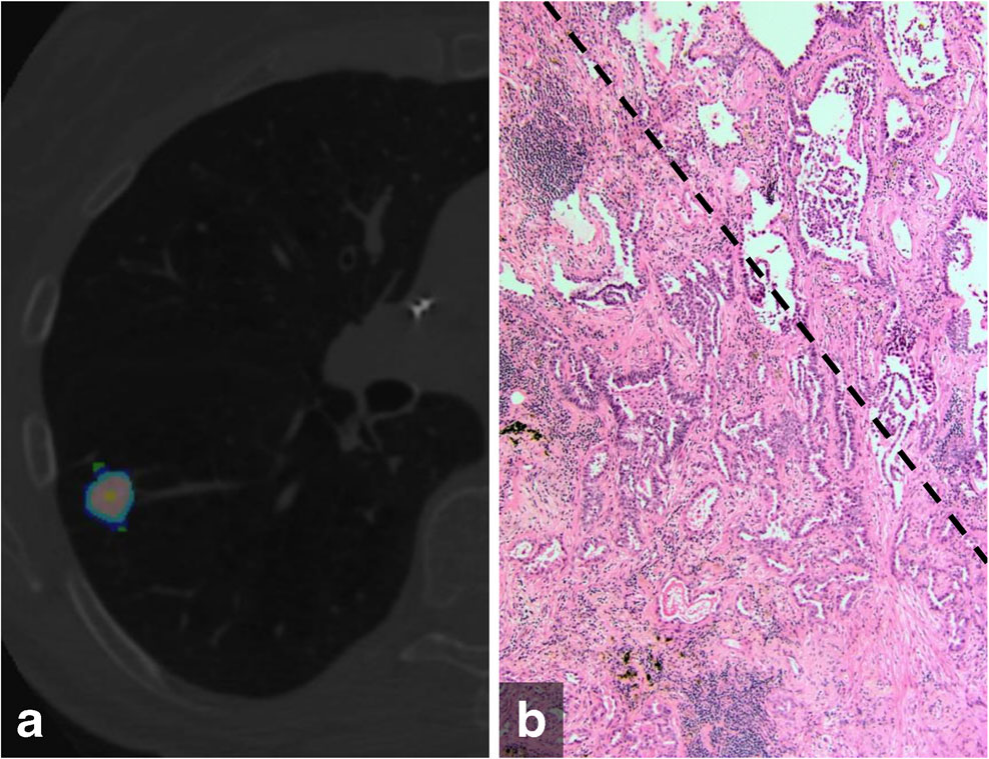
(a) Color-coded CANARY output overlay of minimally invasive adenocarcinoma (blue-green-cyan-pink-yellow) (low risk 55.7%, intermediate risk 44.3%, high risk 0%) (b) histologic image showing the transition zone (dotted line) of peripheral non-invasive lepidic growth pattern (upper right) and the more central invasive acinar pattern component (lower left)(H&E stain, 200x original magnification)

**Fig. 4:**
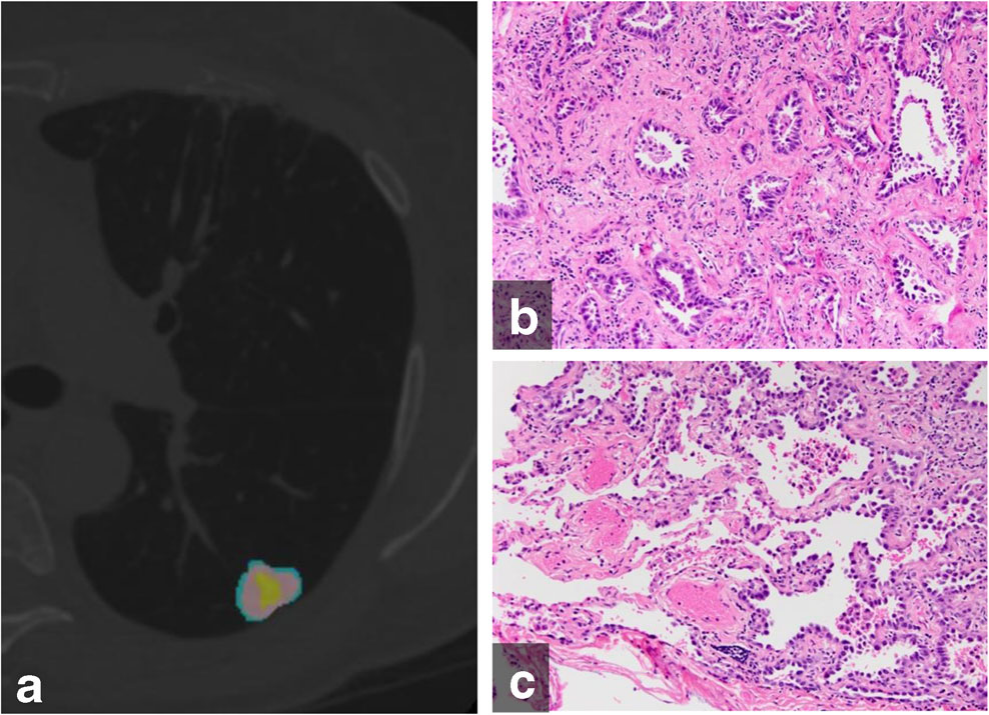
(a) Color-coded CANARY output overlay of invasive adenocarcinoma (yellow-pink-cyan) (low risk 32.0%, intermediate risk 66.9%, high risk 1%)(b) histologic image from the central area of the tumour shows the invasive acinar growth pattern (c) histologic image from the tumour periphery showing non-invasive lepidic growth pattern and adjacent uninvolved lung parenchyma (H&E stain, 200x original magnification)

Examples of CANARY assessment for AIS, MIA, and IAC are displayed in Figs. 2, 3 and 4. The three CANARY components for AIS, MIA, and IACs are displayed in Table 1. The volumes of the low risk CANARY component did not differ significantly between AIS, MIAs, and IACs (*P*=0.408). The volumes of the intermediate risk group were significantly lower in AIS than in MIAs (*P*=0.003), and lower in AIS than in IACs (*P*<0.001). However, the volume difference between MIAs and IACs was not statistically significant (*P*=0.055). The volumes of the high risk group were significantly lower in AIS than in MIAs (*P*=0.040), in AIS than in IACs (*P*<0.001), and in MIAs than IACs (*P*=0.001).

**Table 1:** Volume and percentages of CANARY components for adenocarcinoma in situ (AIS), minimally invasive adenocarcinoma (MIA) and invasive adenocarcinoma (IAC)

	Low risk		Intermediate risk		High risk	
	Volume	Percentage	Volume	Percentage	Volume	Percentage
	(mL)	(%)	(mL)	(%)	(mL)	(%)
AIS, *n*=28	1.1±1.2	78.5±17.6	0.3±0.5	21.5±17.6	0.0±0.0	0.0±0.0
	(0.2,7.1)	(45.2,100.0)	(0.0,2.7)	(0.0,54.8)	(0.0,0.0)	(0.0,0.1)
MIA, *n*=26	1.5±1.7	65.3±27.0	0.9±1.6	32.3±23.5	0.1±0.3	2.4±6.6
	(0.2,7.1)	(15.5,100)	(0.0,8.2)	(0.0,71.0)	(0.0,1.6)	(0.0,29.6)
IAC, *n*=10	2.3±2.8	46.6±22.9	3.9±6.9	49.9±19.8	0.7±1.9	3.4±5.5
	(0.3,7.4)	(20.1,90.0)	(0.1,23.3)	(10.0,66.9)	(0.0,6.3)	(0.0,17.1)

Data are displayed as mean ± standard deviation, together with (range)

The percentages of the low risk CANARY component were significantly higher in AIS than MIAs (*P*=0.034), in AIS than IACs (*P*<0.001), and in MIAs than IACs (*P*=0.024). The percentages of the intermediate risk component were significantly lower in AIS than MIAs (*P*=0.037), in AIS than IACs (*P*<0.001), and in MIAs than IACs (*P*=0.016). The percentages of the high risk group were significantly lower in AIS than in MIAs (*P*=0.037), in AIS than in IACs (*P*<0.001), and in MIAs than IACs (*P*=0.002).

The relations of the size of the invasive focus and the volumes of the intermediate and high risk components were statistically significant (*r*=0.477, *P*<0.001, and *r*=0.471, *P*<0.001, respectively). However, the relation of the size of the invasive focus and the volume of the low risk component was not statistically significant (*r*=0.200, *P*=0.679). The relations between the percentage of the three CANARY components with the size of invasive focus were statistically significant (low risk: *r*=-0.406, *P*=0.005; intermediate risk: *r*=0.407, *P*=0.005; high risk: *r*=0.467, *P*<0.001), respectively.

**Fig. 5:**
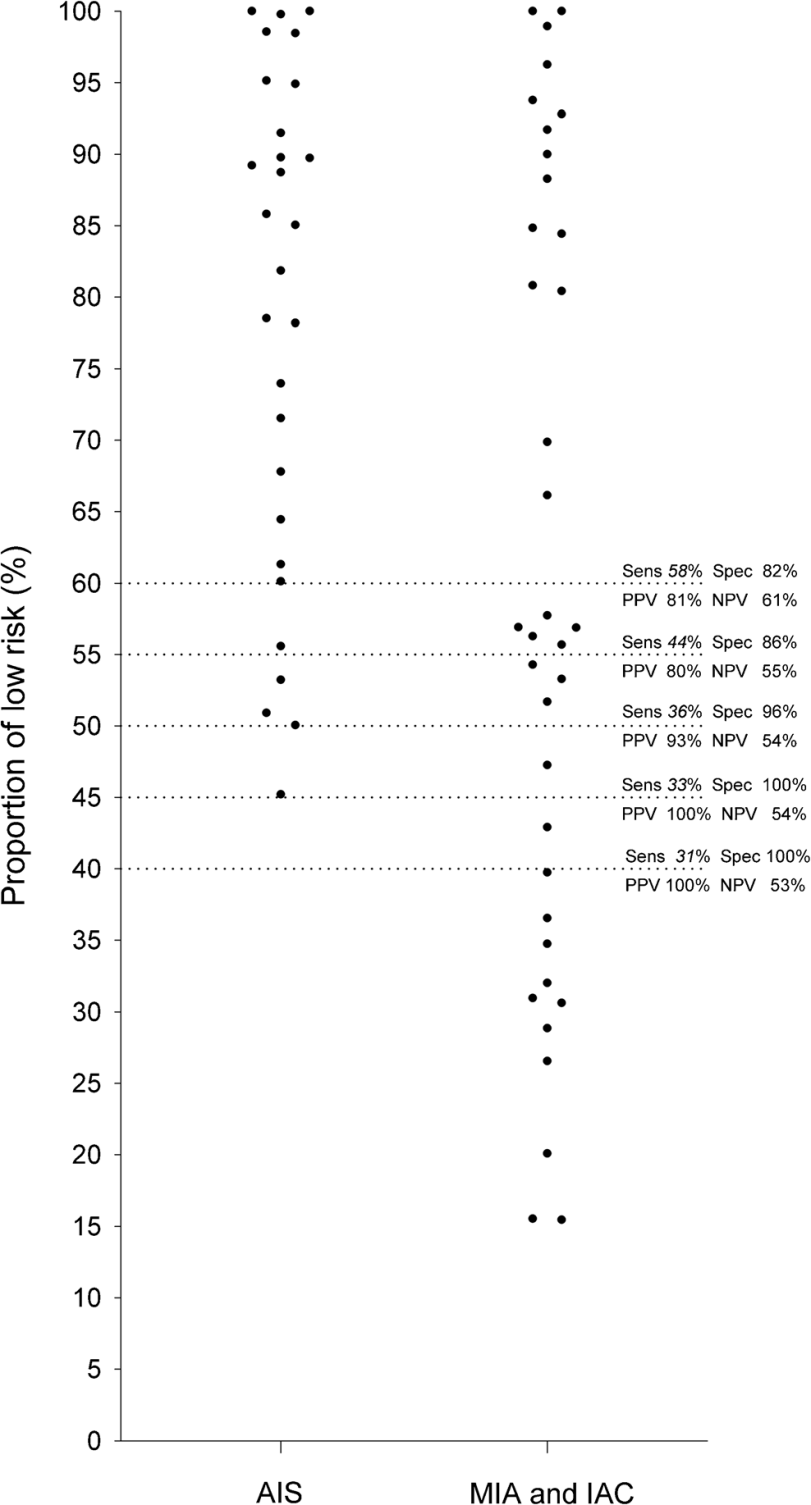
Individual percentages of the CANARY low risk components for non-invasive (adenocarcinoma in situ) and invasive (minimally invasive adenocarcinoma and invasive adenocarcinoma) groups Note.- AIS- adenocarcinoma in situ; MIA - minimally invasive adenocarcinoma: IAC- invasive adenocarcinoma; Sens – Sensitivity; Spec- Specificity; PPV-positive predictive value; NPV – negative predictive value

Individual percentages of the low risk group are displayed in Fig. 5. For each threshold value, sensitivity, specificity, NPV, and PPV are shown in Table 2.

**Table 2:** Sensitivity, specificity, positive predictive value and negative predictive value, together with their (95% confidence intervals) for predefined thresholds of CANARY component percentages

Cutoff	Sensitivity	Specificity	Positive predictive value	Negative predictive value
≤ 40%	30.6% (16.3% - 48.1%)	100.0% (87.7% - 100.0%)	100.0% (71.5% - 100.0%)	52.8% (38.6% - 66.7%)
≤ 45%	33.3% (18.6% - 51.0%)	100.0% (87.7% - 100.0%)	100.0% (73.5% - 100.0%)	53.8% (39.5% - 67.8%)
≤ 50%	36.1% (20.8% - 53.8%)	96.4% (81.7% - 99.9%)	92.9% (66.1% - 99.8%)	54.0% (39.3% - 68.2%)
≤ 55%	44.4% (27.9% - 61.9%)	85.7% (67.3% - 96.0%)	80.0% (56.3% – 94.3%)	54.5% (38.8% - 69.6%)
≤ 60%	58.3% (40.8% - 74.5%)	82.1% (63.1% - 93.9%)	80.8% (60.6% - 93.4%)	60.5% (43.4% - 76.0%)
≤ 65%	58.3% (40.8% - 74.5%)	71.4% (51.3% - 86.8%)	72.4% (52.8% - 87.3%)	57.1% (39.4% - 73.7%)
≤ 70%	63.9% (46.2% - 79.2%)	67.9% (47.6% - 84.1%)	71.9% (53.3% - 86.3%)	59.4% (40.6% - 76.3%)

Individual percentages of intermediate risk group are shown in Supplementary Fig. 1.

Linear regression analysis showed no relation between CANARY component percentages and contrast material administration (*P*=0.331 to 0.664) as well as section thickness (*P*=0.255 to 0.762).

## Discussion

Our study showed that in ACs manifesting as pure ground glass nodules on CT, CANARY software allowed the differentiation of the histological subtypes AIS, MIA and IAC. The analysis software indeed showed statistically significant differences in risk components between these three subtypes. Previously published studies also showed differences in risk components between histological AC subtypes [1–3]. However, these studies included morphologically heterogeneous groups of ACs, whereas our study showed this in a very homogeneous group of ACs. Therefore, to the best of our knowledge, our findings are novel in showing these differences in a homogenous group by exclusively including only ACs manifesting as pure ground glass nodules. This is important because risk assessment of ACs manifesting as pure ground glass nodules is essential for choosing a management approach such as CT follow-up interval, time of biopsy, and selection of an appropriate surgical method [10–15].

In our study, the percentage of low risk components decreased from AIS to MIA and from MIA to IAC. This likely mirrors the consistency of CANARY assessment with histological characteristics of AC subtypes. Simultaneously, the percentage of intermediate risk and high risk components increased from AIS to MIA and from MIA to IAC. Again, this may mirror the higher number of invasive foci in these histological subtypes [16, 17]. Note that in our study, AIS did not show high risk components which should be expected as AIS is by definition a pre-malignant neoplasm without invasive foci [16, 17]. Furthermore, the percentage of high risk components was, in general, also low in MIA and IAC, which may be due to our morphologically homogeneous group of ACs and its similar attenuation profiles [18]. Our study showed that analysis software was able to detect differences even in this very homogeneous group.

Our study also showed that a low risk component threshold of 45% provided a 100% specificity and positive predictive value for invasiveness of ACs manifesting as pure ground glass nodules. At a threshold of 50%, the PPV was still 93% with a specificity of 96% and only a minimal gain in sensitivity. This reflects that below a threshold of 50%, there is a close to 100% likelihood of the nodule being invasive. This further indicates that the PPV for invasiveness is the strength of this software when used in ACs manifesting as pure ground glass nodules.

On the other hand, our results showed a low sensitivity of approximately 30% for histological invasiveness. A previously published pilot study reported a sensitivity of 95.4 to 98.7% and a negative predictive value of 87.5 to 96.8% for a different histological subcategorization of pulmonary adenocarcinomas [1]. Therefore, and because this study also included both, solid and part-solid lesions, the diagnostic performance of these two studies are difficult to compare.

Previous studies reported lesion size to be an important predictor for invasiveness in ACs manifesting as pure ground glass nodules [19, 20]. Lee et al. showed that an overall lesion size of < 10 mm can be used to differentiate between pre-invasive and invasive lesions [21]. In contrast, Liu et al. reported lesions smaller than 10 mm also to be invasive ACs [10]. In our study, we focused on component size rather than on overall lesion size and our results showed a rather weak but statistically significant relation between the size of the invasive focus and the relative percentages of the three CANARY components. This suggests that size can but must not be considered a relevant factor for predicting invasiveness. Moreover, size thresholds are controversial. Therefore, the analysis software could better facilitate in the prediction of invasiveness of ACs. This prediction is important as patients with suspected invasive nodules are more likely to undergo more follow-up examinations, earlier biopsies, and more radical surgical resections [17, 22]. In particular, the software may be used in high risk patients.

Our study has several limitations. First, our study included only surgically resected ACs manifesting as pure ground glass nodules that were surgically resected and histologically proven to be ACs. For this reason, our nodule sample might have been skewed towards larger, morphologically more conspicuous or aggressively behaving ground glass nodules. However, this particular study design warranted a pathologically homogeneous sample of nodules. Prospective study data will be required to confirm our current results. Second, CANARY analysis is a semiautomatic software tool, requiring the manual drawing of regions of interest, a time consuming factor that may limit its clinical use. Third, our study may be limited by the variability in CT protocols. However, at the time of the CT acquisitions, each individual CT scanner was considered to be state-of-the-art. All image data were reconstructed with standard reconstruction algorithms, which have been described to be similar between different scanners [23]. Furthermore, to exclude the influence of contrast media administration and section thickness, we included these variables in our statistical analyses. Fourth, we did not address whether the software is able to differentiate between benign and malign lesions. However, the density reduction algorithm was developed by using only pulmonary nodules that were histologically proven adenocarcinomas of the lung. Although we believe that the differentiation between benign or malign lesions is important in daily routine, the focus of our study was to test the ability of CANARY software to differentiate between histological subtypes of adenocarcinomas manifesting as pure ground glass nodules. Further studies will have to assess whether CANARY can differentiate between benign and malignant lesions.

In conclusion, our study showed that CANARY-based risk stratification of ACs manifesting as pure ground glass nodules allows the differentiation of AIS, MIA and IACs, the histological subtypes of these lesions. Moreover, CANARY software reflects invasiveness in this group of ACs manifesting as pure ground glass nodules using a threshold of 45% or lower of low risk components. While the precise role of CANARY in the work-up of ACs remains to be determined, our study suggests a potential role for this software in suspected ACs manifesting as pure ground glass nodules on CT, notably for patients with high risk of invasiveness.

## Electronic supplementary material

Below is the link to the electronic supplementary material.

ESM 1 (GIF 14 kb)

High resolution image (TIF 1463 kb)
