# Supplementary figures and images for: Software-based risk stratification of pulmonary adenocarcinomas manifesting as pure ground glass nodules on computed tomography
Source: Eur Radiol. 2017 Jul 14;28(1):235–42. doi: 10.1007/s00330-017-4937-2 (PMC5717124; doi:10.1007/s00330-017-4937-2)

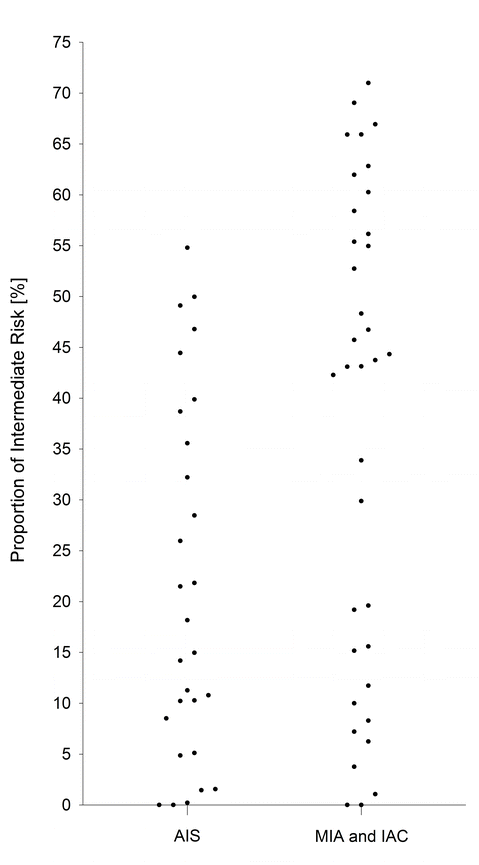

Supplement: Supplementary file 1 — (GIF 14 kb) [file 330_2017_4937_Fig6_ESM.gif]

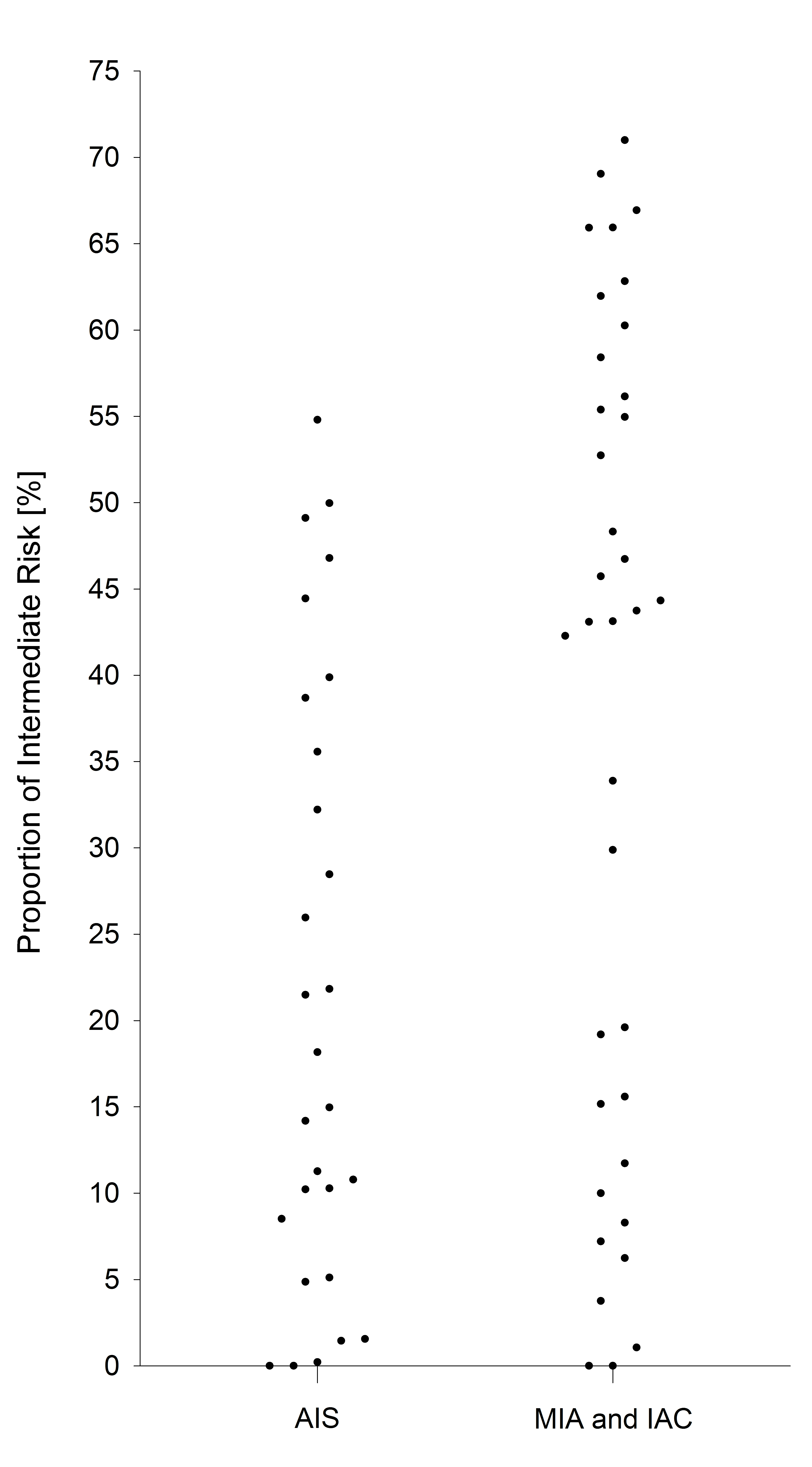

Supplement: Supplementary file 2 — High resolution image (TIF 1463 kb) [file 330_2017_4937_MOESM1_ESM.tif]
